# Networks: On the relation of bi- and multivariate measures

**DOI:** 10.1038/srep10805

**Published:** 2015-06-04

**Authors:** Wolfgang Mader, Malenka Mader, Jens Timmer, Marco Thiel, Björn Schelter

**Affiliations:** 1 Institute of Physics, University of Freiburg, Germany; 2 Freiburg Center of Data Analysis and Modeling, University of Freiburg, Germany; 3 University Medical Center Freiburg, Germany; 4 BIOSS, Center for Biological Signalling Studies, University of Freiburg, Germany; 5 University of Aberdeen, UK

## Abstract

A reliable inference of networks from observations of the nodes’ dynamics is a major challenge in physics. Interdependence measures such as a the correlation coefficient or more advanced methods based on, e.g., analytic phases of signals are employed. For several of these interdependence measures, multivariate counterparts exist that promise to enable distinguishing direct and indirect connections. Here, we demonstrate analytically how bivariate measures relate to the respective multivariate ones; this knowledge will in turn be used to demonstrate the implications of thresholded bivariate measures for network inference. Particularly, we show, that random networks are falsely identified as small-world networks if observations thereof are treated by bivariate methods. We will employ the correlation coefficient as an example for such an interdependence measure. The results can be readily transferred to all interdependence measures partializing for information of thirds in their multivariate counterparts.

Complex systems are ubiquitous. Typically they are represented by networks and also visualized as such. Modern societies rely on the understanding of the dynamics of these networks, for instance, to be able to interfere with them. Examples of complex systems modeled as networks include power grids, traffic systems, climate, or neuronal oscillators.

A network *G*(*V*,*L*) is defined as a set of nodes *V* = {*n*_1_,*n*_2_,…,*n*_*N*_} with |*V*| = *N*, and, in general, an ordered set of links *L*, *L* ⊂ {(*n*_*i*_,*n*_*j*_) ∈ *V* × *V*}, with |*L*| = *D*, where |⋅| denotes the number of elements in a set. The number of links *d*_*i*_ attached to *n*_*i*_ is called the degree of the node. The links of an undirected network can be represented in a symmetric adjacency matrix *A* with entries *a*_*ij*_ = 1 if *l*_*ij*_ ∈ *L*, and 0 otherwise. By collecting the nodes in rows and the links in columns, the *N *× *D* incidence matrix is constructed. It is *b*_*ij*_ = 1 if the *j*-th link is attached to *i*-th node, and zero otherwise.

Here, we are concerned with the typical scenario, that the network itself needs to be inferred from measurements; no prior knowledge of the network topology is available. The nodes are identified with the processes of interest; often but not necessarily the locations of their measurements. The links between them are determined using interdependence measures; the correlation coefficient is among the most frequently applied ones^1^,^2^.

Classifying interdependence measures into two classes, (i) bivariate and (ii) (truly) multivariate approaches, provides the basis for our investigations. Here, we use the notion “multivariate” for the simultaneous analysis of interdependences between three or more processes. We demonstrate that bivariate network reconstruction techniques, if applied naively, fall short in reconstructing the network’s topology as well as its classification, for example as a small world network. We note that, e.g., in directed acyclic graphs an apt combination of bivariate measures can also reveal the correct network topology.

Prior related work can be found in e.g.^1^,^3^,^4^,^5^ The findings presented in these publications are based on data obtained by simulating different models.

In this manuscript, we suggest a mathematical framework that independent of any specific nodal dynamics enables a deeper understanding of network inference. Our analytical results are illustrated and supported by simulations.

## Theory on data-based network inference

In this section, we outline the key differences of bi- and multivariate interdependence measures and the implications for network reconstruction. We also show how the topology resulting from a bivariate measure can be derived from the topology of the respective multivariate one, using the (partial) correction matrix as an example.

### Bivariate and multivariate measures

The key challenge in network inference from measurements is to determine the direct links, i.e. those links that represent a true physical interaction between two nodes. A naive application of a bivariate approach would analyze all pairwise combinations and investigate whether or not there is an interaction. This is suboptimal as indirect links are falsely classified as links in a network. Multivariate interdependence measures, in contrast, exploit all (linear) information of the N observations simultaneously. For various bivariate techniques, multivariate counterparts have been suggested, enabling a direct comparison between the two. The results presented here hold for all multivariate measures whose estimation can be reduced to inverting the matrix of the corresponding bivariate measure^6^ such as the multivariate versions of correlation, cross-spectral analysis^7^, mean phase coherence^8^, and recurrence analysis^9^.

**Figure 1:**
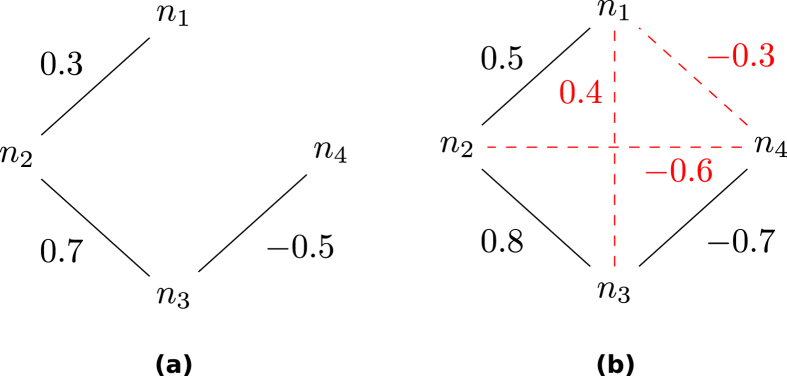
(**a**) Network with only direct links and arbitrarily chosen weights compatible with partial correlation coefficients.(**b**) Fully connected network including indirect links and weights of the corresponding rounded bivariate correlation coefficients.

Taking the network in Fig. 1 (a) as an example, a multivariate measure detects only direct links (black lines). Applying the corresponding bivariate measure, the network in Fig. 1 (b) would result, where the dashed lines indicate indirect links, which are suggested from bivariate analysis.

Weights in Fig. 1 (a) are chosen arbitrarily but compatible with a multivariate correlation analysis. Therefore, the weights can be understood as partial correlation coefficients; those given in Fig. 1 (b) are the corresponding bivariate correlation coefficients. In the following we characterize the relation between the two.

### Bivariate and partial correlation

Let *Z* be an (*s* + *t*)-, *X* an *s*-, and *Y* a *t*-vector valued stationary process with covariance matrix
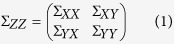
such that *X* and *Y* are a partition of *Z.*

The partial covariance matrix^6^,^10^
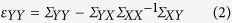
measures the covariance within *Y* after removing the linear effects of *X*. By normalizing *ε*_*YY*_, the partial correlation matrix of *Y*^6^.
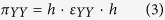
is obtained. The matrix *h* consists purely of the diagonal entries 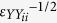, *i* = 1,…, *t* of the *t*-dimensional process *Y*. Partial correlation is a linear multivariate symmetric measure, 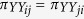, quantifying the strength of the direct connection between *Y*_*i*_ and *Y*_*j*_ conditioned on the linear information of *X*. Often *Y* is chosen to be two dimensional, investigating whether or not there is a link between two processes.

Correlation is normalized covariance, the following line of argument applies to both quantities. It is convenient to start with the covariance matrix ϱ. Given ϱ for the full *s* + *t*-dimensional system, the partial correlation matrix can be obtained by the following steps: (i) matrix inversion^6^
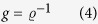


and (ii) a subsequent normalization 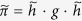, with 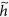 a diagonal matrix and 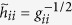, *i* = 1,…, *s* + *t*. To arrive at the partial correlation matrix π, (iii) the off-diagonal elements of π have to be multiplied by −1^6^,^11^,
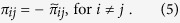


The algorithm given in Eqs. (4)–(5), 10 relates correlation and partial correlation coefficients. It is interesting to note that given this procedure all partial correlation coefficients are obtained in one step; further calculations that would result when applying Eq. (2) are not needed.

## Theoretical results

Reversing the argument given above, we can derive the correlation matrix from the partial correlation matrix. This is exploited in the following to investigate how indirect links emerge when applying bivariate techniques naively.

Any partial correlation matrix can be separated into
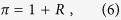
where the off-diagonal entries of *R* are populated with the partial correlation coefficients while its diagonal is zero. Multiplying the off-diagonals by − 1 [cf. Eq.(5)] leads to
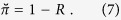


Expanding the matrix inversion
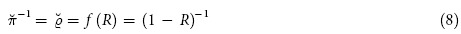
in a Taylor series about *R* = 0 results in
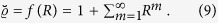
The matrix 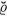 is up to normalization the bivariate correlation matrix. In the first order approximation
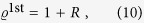


the correlation matrix equals the partial correlation matrix [Eq. (6)]. This shows that all indirect links are encoded in second and higher order terms of the matrix expansion [Eq. (9)]. This fact has key consequences for the nature of indirect links:

- Typically, powers of *R* lead to non-zero values for all entries of ϱ leading to a fully connected network.
- The bivariate correlation coefficients typically differ from the multivariate ones also for the direct interactions.
- If *R* is a nilpotent matrix of degree *z*, the sum in Eq. (9) has only *m* = *z* − 1 terms. Network topologies with a nilpotent matrix *R* of degree 2 yield the same result for the correlation and partial correlation matrix.
- For a directed chain *n*_1_ → *n*_2_ → … → *n*_*N*_ the link between bivariate and multivariate measure can be understood intuitively. For *N* = 4, for instance,
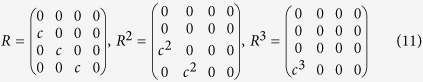
- it becomes obvious, that the indirect links resulting from *R*^2^ are passing one node and *R*^3^ are passing two nodes. Note, that the strengths of the indirect links (|*c*| < 1) scale as *c*^*i*^ where *i* is the order of the link; intuitively it is the shortest path using direct links that manifests this indirect link.
- For an undirected chain, *R* is no longer nilpotent. Still, the lengths of paths inducing indirect links scale with the powers of *R*. Of special interest is the situation of a three node undirected chain

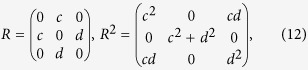


since such open triangles are closed when treated by a bivariate measure. In a local second order approximation, the weight of the indirect link emerging from an open triangle is proportional to the product of the two direct links involved.

In case *c* and *d* are strong direct connections, they have large partial correlation coefficients compared to the average partial correlation coefficient appearing in the network. The corresponding indirect connection is then likely to have a large correlation coefficient, compared to the average correlation coefficient. If a threshold is used to select relevant links, a strong but indirect link might be selected, while a weak direct link which would represent a true physical connection in the network is missed. The reason for the so called transitivity property of bivariate measures^1^,^4^ is therefore founded in the fact, that indirect links constituted by only two direct ones are introduced already in the second order Taylor approximation; making their magnitude comparable to correlation coefficients of direct links.

In the following paragraphs, the effects of these properties are illustrated in a simulation study. The focus is on the effect of naive bivariate analysis onto the network topology and network classification. We emphasis again that our simulations are free of any assumptions about nodal dynamics, which demonstrates the universality of our framework.

## Simulation setup and results

Using the framework introduced in the last section, a simulation study is conducted. We compare the outcome of correlation coefficient and partial correlation coefficient when used to infer links in different network topologies. To this end, a regular network and two versions of random networks are investigated. In the first version, all partial correlation coefficient have the same value, in the second version, those values vary. The section is completed by a result showing that random networks are falsely classified as small world networks in the frame of bivariate interdependence measures.

### Estimating links in regular networks

The first system investigated is a regular network with *N* = 80 nodes. In this network, each node is connected to its nearest neighbors only, resulting in a ring with *D* = 80 direct links. To demonstrate that indirect links of increasing length correspond to increasing orders of the Taylor series [Eq. (9)], all nodes must be identical. Hence, all links have to share the same strength. A correlation matrix must be positive semidefinite. Derived from Sylvester’s criterion, such a matrix can be obtained by ensuring, that the matrix is Hermitian, has real and non-negative diagonal elements, and is diagonal dominant. The first two criteria are naturally meet by correlation matrices, while the last one must be cared for. Since in the regular network considered here, each node has degree 2, the value of the partial correlation coefficient was chosen to 0.49, which is near the largest possible value (2⋅0.49 < 1).

**Figure 2:**
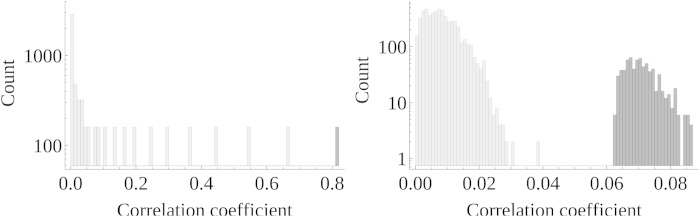
To all links in a regular (left) and a random (right) network of 80 nodes each, the same weight representing a partial correlation coefficient was attached. The corresponding correlation coefficients are calculated and summarized in these histogram. (left) Due to the regularity of the network, the correlation coefficients cluster, where the 160 largest represent the direct (dark gray) the other the indirect links (light gray). (right) Because the network topology is random, nodes differ in the number of links attached to them. This influences the values of the correlation coefficients and destroys the strict clustering with respect to path length. Direct links (dark gray) exhibit larger correlation coefficients than indirect ones (light gray). In both networks a threshold allows to separate direct and indirect links.

The corresponding bivariate correlation coefficients, indicating direct and indirect links, are obtained from the partial correlation matrix using the framework introduced above[Eqs. (6) –(9), 12, 13, 14]. The expected clustering of the correlation coefficients with respect to the length of their constituting path is visible from their histogram [Fig. 2 , left]. The 80 “truly” direct links are shown in dark gray; these are the strongest links as expected from our framework. In light gray, the correlation coefficients of indirect links are presented. The right most (light gray) cluster refers to first order indirect links *n*_*i*_—*n*_*i*+2_, and so on. In this scenario, thresholding the correlation coefficients would provide the correct network topology if the threshold were chosen “correctly” between 0.67 and 0.8.

### Estimating links in random networks

The second system investigated assumes an Erdös-Rényi network with *N* = 80 nodes, and *D* =  = 351 links. Using about *N*log(*N*) links guarantees no unconnected node in the network^12^,^13^. As in the last simulation, all links share the same partial correlation value in the partial correlation matrix describing the network. For the matrix to be diagonal dominant, and hence positive definite, this value must be chosen smaller than 1/*d*_Max_, where *d*_Max_ is the maximum node degree in the network. As predicted by our framework, the orders of the Taylor expansion are no longer preserved in the histogram of correlation coefficients [Fig. 2 , right]; but still all correlation coefficients representing indirect links (light gray) are smaller than the ones representing direct ones (dark gray). Again a threshold can be found which separates direct and indirect links; this allows for the correct inference of the true network topology.

### Connections of varying strength

**Figure 3:**
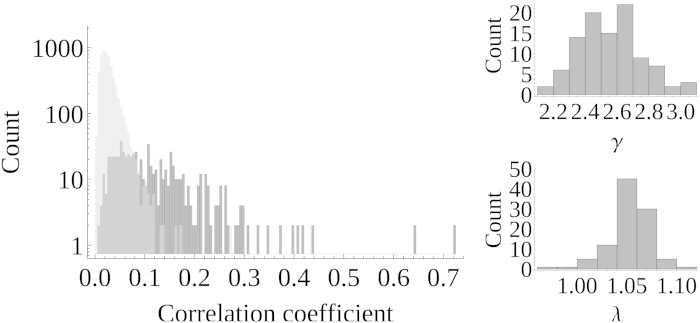
(left) In an 80 nodes random network, different weights representing partial correlation coefficients are attached to its links. The histogram summarizes the corresponding correlation coefficients. In dark gray, the correlation coefficients of direct links, in light gray the ones of indirect links are presented. Because their values overlap, applying a threshold to correlation coefficients will report a mixture of direct and indirect links and hence a wrong network topology; wrong conclusion about e.g. the small world property of a network are drawn. (right) This is demonstrated by the histograms of the small world indicators γ and λ which are calculated from 100 realizations of a random network in which the largest 351 correlation coefficients were used to establish its links. Since γ > 1 and λ ~ 1 in all cases, every random network observed through correlation coefficients was falsely classified as small world.

When dealing with random networks and varying strengths of links, a more complicated scenario emerges. To obtain the partial correlation matrix, we start with the incidence matrix of an Erdös-Rényi network of *N* = 80 nodes and *D* = 351 links. The strengths of links are drawn from the uniform distribution over the interval [0,1]. The partial correlation matrix is obtained as the normalized matrix square of this weighted incidence matrix. According to Eq. (12), a weak direct link can have a smaller correlation coefficient than an indirect link resulting from two strong direct links. The situation is visualized in Fig. 3 on the left. Again, correlation coefficients of direct links are shown in dark gray, correlation coefficients of indirect ones in light gray. Since their distributions overlap, they are not separable by a threshold.

When estimating links from measurements, the underlying network is unknown. Typically, the network exhibits non-regular topology and varying link strengths. As demonstrated, thresholding bivariate interdependence measures fails in these typical cases to reveal the correct network topology.

To demonstrate that this has broad implications for network inference, the consequences of using bivariate interdependence measures for inferring the small world property is investigated as an example.

### Small-world network

The local clustering coefficient
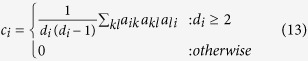


and the average shortest path length
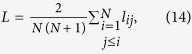


characterize the network topology on the local and global scale. In a network exhibiting the small world property, the mean clustering coefficient 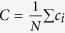 is high while the average shortest path length *L* scales with the number of nodes *N* as *L*(*N*) ≤ log *N*^12^,^14^,^15^. According to Eq. (14), the average shortest path lengths would be infinity in networks possessing at least one unconnected node. Similar to^15^ the path lengths of pairs of nodes involving an isolated node are excluded when evaluating Eq. (14).

Since in applications the scaling of *L*(*N*) is often not accessible, two proxy indicators 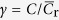 and 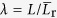 are employed. The mean values 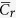 and 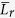 are obtained from an ensemble of random networks. A network is said to be small world if λ ≈ 1 and γ > 1 holds^1^,^15^.

From the partial correlation matrix of a random network (*N* = 80), generated in the same way as described in the last section, the corresponding correlation matrix is calculated. A threshold is applied to the correlation coefficients to obtain the *D* = 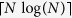 = 351 strongest links. This network is tested for small worldness. In order to get a statistically meaningful result, a Monte Carlo simulation of 100 different realizations of the partial correlation matrix is carried out.

The histograms of the resulting λ and γ are presented in Fig. 3 on the right. For all realizations, γ > 1 [Fig. 3, top] while λ ≈ 1 [Fig. 3, bottom]. Therefore, all random networks are falsely classified to be small world. The classification is based on the bivariate networks alone. The explanation of the result is the transitive property for which the mathematical foundation was presented in our framework in Eqs. (9) and (12) . Hence, this result applies for all networks in which links are estimated by a naive application of a bivariate interdependence measure irrespective of the dynamics observed, the measurement technique used, or the specific bivariate measure employed.

## Conclusion

In this manuscript, we inferred the nature of indirect links introduced by bivariate interdependence measures. We developed a mathematical framework that we motivated for correlation analysis. To this end, the inverse partial correlation matrix was expanded into a Taylor series. We demonstrated that, in general, bivariate measures are not able to reveal the true network topology from measurements; indirect links which have no underlying physical connection in the observed system are incorrectly inferred, altering the network topology.

The clustering coefficient is strongly affected by investigating networks based on the naive application of bivariate approaches. It influences high-level characterizations; a random network is e.g. falsely classified as small world in this context. The reason for this is the tendency of bivariate measures to close open triangles, i.e., the transitivity property.

Multivariate measures promise to overcome this limitation. Only links which are direct given the information of all measurements give rise to values of the interdependence measure which are not compatible with zero. Accordingly, a statistically meaningful critical value for a given level of significance can be obtained, rendering the choice of an arbitrary threshold unnecessary. Surrogate or bootstrapping approaches present alternatives to analytically derived critical values. Therefore, links reported by a multivariate measure assuming observation of all relevant processes are supposed to represent true connections, constituting the correct network topology. Since such reconstructed networks do not suffer from an increased clustering coefficient network classification can be done successfully.

## Additional Information

**How to cite this article**: Mader, W. *et al.* Networks: On the relation of bi- and multivariate measures. *Sci. Rep.* **5**, 10805; doi: 10.1038/srep10805 (2015).

## References

[b1] Hlinka J., Hartman D. & Palus M. Small-world topology of functional connectivity in randomly connected dynamical systems. Chaos 22, 033107 (2012). 23020446 10.1063/1.4732541

[b2] Tewarie P. *et al.* Functional brain networks: Linking thalamic atrophy to clinical disability in multiple sclerosis, a multimodal fMRI and MEG Study. Hum. Brain Mapp. 36, 603–618 (2015). 25293505 10.1002/hbm.22650 PMC6869443

[b3] Bialonski S., Wendler M. & Lehnertz K. Unraveling Spurious Properties of Interaction Networks with Tailored Random Networks. PLoS One 6, e22826 (2011). 21850239 10.1371/journal.pone.0022826 PMC3151270

[b4] Zalesky A., Fornito A. & Bullmore E. On the use of correlation as a measure of network connectivity. NeuroImage 60, 2096–2106 (2012). 22343126 10.1016/j.neuroimage.2012.02.001

[b5] Zerenner T., Friederichs P., Lehnertz K. & Hense A. A Gaussian graphical model approach to climate networks. Chaos 24, 023103 (2014). 24985417 10.1063/1.4870402

[b6] Lauritzen S. Graphical Models (Oxford University Press, 1996).

[b7] Dahlhaus R. Graphical interaction models for multivariate time series. Metrika 51, 157–172 (2000).

[b8] Schelter B., Winterhalder M., Dahlhaus R., Kurths J. & Timmer J. Partial Phase Synchronization for Multivariate Synchronizing Systems. Phys. Rev. Lett. 96, 208103 (2006). 16803212 10.1103/PhysRevLett.96.208103

[b9] Nawrath J. *et al.* Distinguishing Direct from Indirect Interactions in Oscillatory Networks with Multiple Time Scales. Phys. Rev. Lett. 104, 038701 (2010). 20366687 10.1103/PhysRevLett.104.038701

[b10] Brillinger D. *Time Series: Data Analysis and Theory*. Classics in Applied Mathematics (Society for Industrial and Applied Mathematics , 2001).

[b11] Whittaker J. Graphical Models in Applied Multivariate Statistics (Wiley, 1995).

[b12] Boccaletti S., Latora V., Moreno Y., Chavez M. & Hwang D. Complex networks: Structure and dynamics. *Phys. Rep.* 424, 175–308 (2006).

[b13] Erdös P. & Rényi A. On random graphs. Publ. Math. Debrecen 6, 290–297 (1959).

[b14] Watts D. & Strogatz S. Collective dynamics of ‘small-world’ networks. Nature 393, 440–2 (1998). 9623998 10.1038/30918

[b15] Bialonski S., Horstmann M. & Lehnertz K. From brain to earth and climate systems: Small-world interaction networks or not? Chaos 20, 013134 (2010). 20370289 10.1063/1.3360561

